# Dietary Patterns Derived Using Exploratory and Confirmatory Factor Analysis are Stable and Generalizable Across Race, Region, and Gender Subgroups in the REGARDS Study

**DOI:** 10.3389/fnut.2014.00029

**Published:** 2015-01-19

**Authors:** Suzanne E. Judd, Abraham J. Letter, James M. Shikany, David L. Roth, P. K. Newby

**Affiliations:** ^1^Department of Biostatistics, School of Public Health, University of Alabama at Birmingham, Birmingham, AL, USA; ^2^Division of Preventive Medicine, School of Medicine, University of Alabama at Birmingham, Birmingham, AL, USA; ^3^Center on Aging and Health, Johns Hopkins University, Baltimore, MD, USA; ^4^Department of Pediatrics and Program in Graduate Medical Nutrition Sciences, Boston University School of Medicine, Boston, MA, USA; ^5^Department of Epidemiology, Boston University School of Public Health, Boston, MA, USA; ^6^Program in Gastronomy, Culinary Arts, and Wine Studies, Boston University Metropolitan College, Boston, MA, USA

**Keywords:** stroke, race, region, dietary patterns, nutritional epidemiology, factor analysis

## Abstract

**Background:** Examining diet as a whole using dietary patterns as exposures is a complementary method to using single food or nutrients in studies of diet and disease, but the generalizability of intake patterns across race, region, and gender in the United States has not been established.

**Objective:** To employ rigorous statistical analysis to empirically derive dietary patterns in a large bi-racial, geographically diverse population and examine whether results are stable across population subgroups.

**Design:** The present analysis utilized data from 21,636 participants in the Reasons for Geographic and Racial Differences in Stroke (REGARDS) study who completed the Block 98 food frequency questionnaire. We employed exploratory factor analysis and confirmatory factor analyses on 56 different food groups iteratively and examined differences by race, region, and sex to determine the optimal factor solution in our sample.

**Results:** Five dietary patterns emerged: the “Convenience” pattern was characterized by mixed dishes; the “Plant-based” pattern by fruits, vegetables, and fish; the “Sweets/Fats” pattern by sweet snacks, desserts, and fats and oils; the “Southern” pattern by fried foods, organ meat, and sweetened beverages; and the “Alcohol/Salads” pattern by beer, wine, liquor, and salads. Differences were most pronounced in the Southern pattern with black participants, those residing in the Southeast, and participants not completing high school having the highest scores.

**Conclusion:** Five meaningful dietary patterns emerged in the REGARDS study and showed strong congruence across race, sex, and region. Future research will examine associations between these patterns and health outcomes to better understand racial disparities in disease and inform prevention efforts.

## Introduction

Assessing dietary differences within a population can be challenging and may involve examining nutrients, foods, or patterns of food intake (1). When further considering dietary patterns as the main exposure of interest, there are *a priori* [e.g., Mediterranean diet score (2), Healthy Eating Index (3)] and *a posteriori* (e.g., factor and cluster analysis) methods (4). Data reduction techniques such as factor analysis are empirical methods commonly used to describe dietary patterns in a population, as they are data-driven and represent what people actually eat (5). This allows for the heterogeneity of diet across population subgroups to be explored and quantified in a scientific manner. Many groups have derived dietary patterns using factor analysis and shown meaningful associations with chronic disease (6, 7). In general, reproducibility of dietary patterns has been observed across studies. Usually, a relatively healthy pattern of eating characterized by a wide variety of plant-based foods (often identified as “healthy” or “prudent”) and a less healthy pattern categorized by excess sugars, fats, and processed foods (often identified as “Western” or “convenience”) have been identified; traditional, alcohol, and sweets patterns are also commonly observed (6). However, when heterogeneous populations with a broader mix of racial, ethnic, and socio-economic groups have been included in the study, unique patterns have been observed. For example, in the multi-ethnic study of atherosclerosis (MESA), more nuanced diet patterns were identified, including a predominantly vegetarian pattern (7).

The REasons for Geographic and Racial Differences in Stroke (REGARDS) is a large study designed to identify factors associated with racial and regional differences in stroke in the United States (US). Diet has been hypothesized to be one of the key factors explaining these differences (8). We previously demonstrated that both race and region are associated with nutrient differences in REGARDS (9, 10). Our objective in this study was to employ rigorous statistical analyses including exploratory and confirmatory factor analysis (CFA) to empirically derive dietary patterns in a large bi-racial, geographically diverse population and examine whether results are stable and generalizable across population subgroups. We hypothesized that we would observe meaningful dietary patterns that reflected identifiable and unique eating behaviors consistent with the extant literature (e.g., healthy/prudent, less healthy/Western, high in sugar, high in alcohol, and based on traditional foods) from previous studies.

## Experimental Methods

### Study design

The REGARDS study is a population-based random sample of black and white individuals over age 45 years, designed to identify causes of racial and geographic differences in stroke incidence. The methods have been previously described in detail (11). Briefly, 30,239 people were recruited using commercially available lists from Genesys, Inc. (Daly City, CA, USA), which is the same list used by the Behavioral Risk Factor Surveillance Systems (BRFSS) in the US. The study was designed to oversample black participants and people residing in the stroke belt, a region of the country at particularly high risk for stroke (North Carolina, South Carolina, Georgia, Alabama, Mississippi, Tennessee, Arkansas, and Louisiana). The University of Alabama at Birmingham serves as the primary site for data collection and study coordination. Participants were initially contacted through a mass mailing to inform them of an upcoming phone call. REGARDS staff then conducted a 45-min telephone interview to collect data on demographics, socio-economic status, risk factor characterization, and medical history. The telephone response rate was 33% and cooperation rate was 49%, similar to other cohort studies (12). Following the telephone call, a trained health professional went to the participant’s home to obtain written consent and collect blood and urine specimens. They also measured blood pressure, waist circumference, height, and weight and performed an electrocardiogram that was sent to a central reading facility. This study was conducted according to the guidelines laid down in the Declaration of Helsinki, and all procedures involving human participants were approved by the Institutional Review Board at all participating universities. Written and verbal informed consent was obtained from all participants.

### Dietary assessment

When the health professional was finished with the examination portion of the visit, s/he provided the participant with a series of forms that were to be self-administered and sent back to the REGARDS Coordinating Center at the University of Alabama at Birmingham. One of the forms left in the home was the Block 98 food frequency questionnaire (FFQ). The Block FFQ (www.Nutritionquest.com) has been validated for most nutrients using multiple diet records (13). Different versions of this questionnaire have been studied extensively and validated in diverse populations (14). The Block 98 version developed by Block Dietary Data Systems (Berkeley, CA, USA) and distributed by NutritionQuest is an 8-page paper-and-pencil form with more than 150 multiple-choice questions based on 107 food items that can be completed in about 30–40 min. Useable FFQ data were available for 21,636 (72%) participants: 17% did not return FFQ, 3% returned a blank FFQ, 5% did not answer at least 85% of questions, and 3% had biologically implausible caloric intake. Women, white participants, and those who graduated college were most likely to return the FFQ (9, 10). Participants were asked to report on diet over the past year. Pictures were provided to help in identifying portion sizes. Participants mailed the completed FFQ back to the REGARDS Coordinating Center where it was scanned and double verified. The results were then sent to NutritionQuest for scoring, which included a data set that provided the number of grams per day (g/day) for each line item on the FFQ.

We constructed 56 food groups using the original 107 individual FFQ line item variables (gram/day) based on culinary use (e.g., separating high- and low-fat milk) and nutrient similarities, as well as previous studies (6). For example, one item is “beverages containing some juice like Hi-C.” We grouped this item with sugar-sweetened beverages due to nutritional content. When we considered potatoes, fish, and chicken, we separated out the fried items from the non-fried items due to the high-fat content of fried foods and expected differences of use across populations. Some items like “Chinese food” were left as a stand-alone food group due to the uniqueness of the item. Additionally, we created multiple categories of vegetables to preserve regional variability in intakes. Coffee and tea were retained in separate groups as the two are often consumed in different manners across the US. Initially, 58 food groups were created, but evaluation of communality and zero values identified four groups for further consideration and regrouping: the diet shakes/meal replacement variable was eliminated due to extremely low consumption; the breakfast/power bars group was merged into the sweet breakfast foods group; and the low-fat dairy and milk alternative groups were retained as separate groups given their prominence in the US diet. This resulted in 56 final food groups used in the factor analyses (Table 1).

**Table 1:** **Food group descriptions (*n* = 56)**.

Food group name	Individual foods
100% fruit juice	Orange juice, fruit juice
Added fats	Shortening, lard, vegetable oil, olive oil, gravy, mayonnaise
Beans	Baked beans, refried beans, tofu, meat substitutes
Beer	Beer including light beer and non-alcoholic
Bread	White bread, biscuits, bagels, cornbread
Bread – whole grain	Dark bread
Butter	Butter
Candy	Candy (not chocolate)
Cereal	Cold cereals and cooked cereals
Cereal – high fiber	Bran and high fiber cereals
Chinese food	Chinese dishes
Chocolate	Chocolate
Coffee	Coffee
Condiments	Salsa, ketchup, mustard, barbecue sauce
Desserts	Cookies, cakes, pies
Eggs and egg dishes	Eggs
Fish	Non-fried fish, tuna
Fried food	Fried chicken, fried fish
Fried potatoes	French fries
Fruit	Fruits
High-fat dairy	Cheese, cream, ice cream
Liquor	Liquor
Low-fat dairy	Low-fat cheese, ice cream
Margarine	Margarine
Mexican dishes	Tacos, burritos
Milk alternatives	Non-dairy creamer, rice milk, soy milk
Milk – high-fat	Whole and 2% milk
Milk – low-fat	Non-fat and 1% milk
Miscellaneous sugar	Jelly, jam, syrup, sugar in coffee/tea
Mixed dishes with meat	Mixed dishes with beef, pork, or chicken; chili with beans
Organ meat	Liver, gizzard, neckbones, chitlins
Pasta dishes	Spaghetti, other pasta, macaroni and cheese, other cheese dishes
Pizza	Pizza
Potatoes	White potatoes, baked or mashed but not fried
Poultry	Chicken (not fried)
Processed meats	Hot dogs, bacon, sausage, ham, lunch meat
Food group name	Individual foods
Red meat	Beef, hamburger, pork, ribs, veal
Refined grains	Rice, tortillas, crackers
Salad dressing/sauces	Salad dressing
Salty snacks	Salty snacks, chips, popcorn
Seeds, nuts	Peanuts, other nuts, peanut butter
Shell fish	Oysters and shellfish
Soda	Soft drinks
Soup	Vegetable, bean, lentil, and other soups
Sugar-sweetened beverages	Drinks with sugar added (kool aid) or containing some juice (HI-C)
Sweet breakfast foods	Pancakes, waffles, donuts, pastries, breakfast/power bars
Tea	Tea, iced tea
Vegetable – cruciferous	Broccoli, coleslaw, cabbage, greens, collards
Vegetable – dark yellow	Sweet potatoes
Vegetable – green leafy	Green salad, spinach
Vegetable – other	Carrots, corn, green beans, peas, other vegetables
Vegetable – tomato	Tomatoes, tomato juice, vegetable juice
Vegetable mixed dishes	Vegetable stew
Water	Water
Wine	Wine
Yogurt	Yogurt including frozen yogurt

### Dietary pattern derivation and statistical analyses

We first used principal components analysis (PCA), an exploratory factor analysis (EFA), to derive dietary patterns (15). To ensure that the resulting patterns were valid and could be replicated, we used a random split sample method and divided the sample in half as has been done in similar studies of large sample size (15). There were ample numbers in each group for the two analyses (*n* = 10,818 for each group). To guide factor selection, we examined solutions between two and six component vectors for interpretability across samples. We also used a scree plot to examine the variance explained. In part guided by the scree plots and interpretability of the derived factors, we employed an eigenvalue cutpoint of approximately 1.5. Food group loadings were evaluated for communality (sum of squared factor loadings for a group); groups with many zero values (more than 50%), as well as low communality (<0.1) of factor loadings, were considered for removal; however, no such groups were removed from the final solution. PCA was then repeated using the 56 food groups to choose the number of factors to retain in subsequent steps. Factor loadings were calculated after a varimax rotation, and factor scores were derived by multiplying each factor loading by the corresponding food group value for the individual and then summing across food groups to determine the participant’s factor score for each pattern.

One of the goals of this study was to rigorously examine whether our factors should be derived separately for population subgroups (i.e., geographic region of residence, race, and gender) or whether we could utilize patterns in the whole sample. As such, the half-sample was stratified and we conducted three separate PCA analyses by region focusing specifically on the US stroke belt (southeastern US stroke belt/non-belt), gender (male/female), and race (black/white) (15). Coefficients of congruence were determined for each stratification pair for each of the factor number solutions. Congruence close to 1 was deemed excellent and above 0.5 was considered acceptable. The final number of factors retained was chosen based on the solution providing the optimal congruence across region, gender, and race.

Confirmatory factor analysis was utilized on the second half-sample to independently validate the findings from the PCA analyses. Because it can be difficult for CFA models to converge when many nuisance variables (i.e., variables with low factor loadings) are included, only food groups with absolute value loadings >0.20 for a given factor were included in the initial CFA model. Root mean square error of approximation (RMSEA) and comparative fit index (CFI) were evaluated as additional food groups with small loadings were dropped from the model in order to achieve greater parsimony, and to determine if the final solution from PCA was acceptable. Based upon our CFA results and initial EFA, PCA with varimax rotation was then repeated on the whole sample using the five-factor solution in order to obtain factor scores for each individual.

Mean factor scores were calculated for the demographic variables of age, race, gender, region, income, and education and were tested for significance differences across groups using *t*-test for two levels and ANOVA when more than two. PCA, *t*-tests, and ANOVA were performed using SAS 9.2 (SAS Institute Inc., Cary, NC, USA). CFA was performed using MPlus 6.1 (Los Angeles, CA, USA). Microsoft Excel 2007 (Redmond, WA, USA) was used for determination of communality and coefficients of congruence.

## Results

Reviews of the scree plot (Figure S1 in Supplementary Material) and eigenvalues from PCA suggested that a three- to six-factor solution was the best fit to the data, so each of these solutions were evaluated in subsequent stratified PCA steps using the half-sample as described. We characterized congruence as “excellent” when the smallest coefficient was >0.8, “good” between 0.65 and 0.8, “acceptable” between 0.5 and 0.65, and “poor” <0.5. PCA stratified by region of residence revealed excellent congruence for the four- and five-factor solutions, and acceptable congruence for the three- and six-factor solutions, as suggested by Ref. (16). PCA stratified by gender had good congruence for the five- and six-factor solutions and poor congruence for the three and four-factor solutions. PCA stratified by race had acceptable congruence in the five-factor solution, but poor congruence for the other three (Table 2). After examination for interpretability by the investigators and discussion of the results, the five-factor solution was ultimately selected as it was acceptable in all three stratified scenarios and patterns appeared interpretable, meaningful, and consistent to some degree with extant literature (7, 17).

**Table 2:** **Factor congruence across the five-factor solution by region, race, and gender^a^**.

	Factor number	Factor number	Congruence coefficient
**Region**	**Belt**	**Non-belt**	

	1	3	0.886
	2	2	0.949
	3	1	0.859
	4	5	0.845
	5	4	0.851

**Gender**	**Men**	**Women**

	1	3	0.809
	2	1	0.963
	3	2	0.915
	4	5	0.765
	5	4	0.770

**Race**	**Black**	**White**

	1	3	0.801
	2	2	0.910
	3	1	0.781
	4	5	0.638
	5	4	0.574

*^a^A congruence coefficient above 0.5 is considered to be acceptable, meaning that the patters are similar by stratifying variable*.

**Figure 1:**
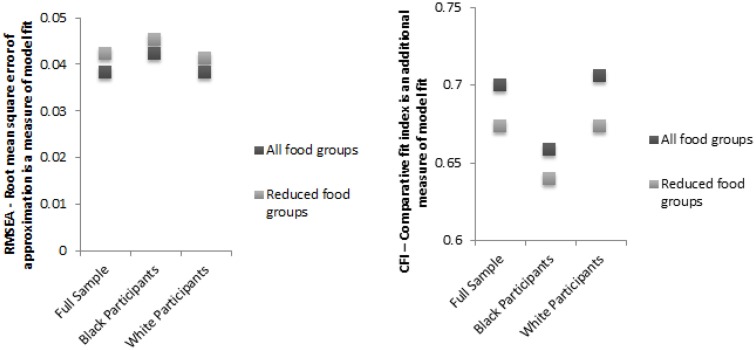
**Sequential model results from confirmatory factor analysis demonstrating the five-factor solution appropriate for the REGARDS population**. Confirmatory factor analysis was used in the second half of the population to verify that the models identified in the exploratory factor analysis could be replicated. All food groups were used for the first analysis and in the second, foods with many zero loadings were removed to verify model fit. Analysis was conducted separately in black participants and white participants and also in the full population.

In addition, CFI and RMSEA from the CFA both provided a good estimate of model fit. Specifically, CFA results for the second half-sample using the five-factor solution were very good, with RMSEA values below 0.05 (Figure 1). Removing food groups with low factor loadings from the model increased the RMSEA slightly but did not cross the 0.05 threshold. Race-specific analyses also resulted in acceptable RMSEA values. Together, these statistics suggested that all models were acceptable and that further removal of food groups would not substantially improve model fit.

Final factor loadings were determined using PCA with varimax rotation of five-factors on the full sample (Table 3). We assigned names to our patterns based both on the factor loadings that contributed most highly to each pattern alongside our knowledge of the general dietary pattern literature (6). Factor 1 loaded heavily on mixed dishes with meat, pasta dishes, Mexican dishes, pizza, red meat, soup, fried potatoes, and Chinese dishes. This group represented mixed dishes of all kinds and various fast foods and was named the “Convenience” dietary pattern. Factor 2 had high factor loadings for cruciferous, green leafy, dark yellow, and other vegetables, as well as fruits, beans, and fish and was named the “Plant-based” pattern. Factor 3 loaded on miscellaneous sugar, desserts, bread, sweet breakfast foods, chocolate, candy, solid fats, and oils and was named the “Sweets/Fats” pattern. Factor 4 loaded heavily on added fats, eggs, fried food, organ meats, processed meats, and sugar-sweetened beverages. Since this diet is similar to the culinary pattern observed in the Southeastern US, this pattern was named the “Southern” pattern. Factor 5 loaded highly on salad dressing, green leafy vegetables, tomatoes, wine, butter, and liquor. We named it the “Alcohol/Salads” pattern because it had the highest factor loadings for wine, liquor, and beer along with leafy greens and salad dressing.

**Table 3:** **Final factor loadings derived in the entire REGARDS population (showing only those with absolute value >0.20 for simplicity)**.

	Convenience	Plant-based	Sweets/fats	Southern	Alcohol/salads
100% fruit juice		0.25			
Added fats			0.40	0.38	0.25
Beans	0.36	0.38			
Beer					0.23
Bread			0.47	0.37	
Bread – whole grain		0.30			
Butter					0.32
Candy			0.40		
Cereal		0.38			−0.20
Cereal – high fiber		0.24		−0.25	
Chinese food	0.44				
Chocolate			0.46		
Coffee			0.22		0.30
Condiments	0.25		0.31		0.29
Desserts	0.20		0.53		−0.17
Eggs and egg dishes				0.42	0.29
Fish	0.27	0.38			0.21
Fried food	0.24			0.56	
Fried potatoes	0.37		0.28	0.16	
Fruit		0.58			
High-fat dairy			0.37		0.21
Liquor					0.31
Low-fat dairy		0.20			
Margarine			0.37		
Mexican dishes	0.48				
Milk alternatives					
Milk – high-fat			0.18	0.24	
Milk – low-fat				−0.42	
Miscellaneous sugar			0.54		
Mixed dishes with meat	0.61				
Organ meat				0.47	
Pasta dishes	0.59				
Pizza	0.45		0.20		
Potatoes	0.36		0.26		
Poultry	0.29	0.31			
Processed meats	0.25		0.26	0.45	0.22
Red meat	0.45			0.26	0.26
Refined grains	0.31		0.20	0.20	
Salad dressing/sauces		0.30			0.55
Salty snacks	0.32		0.30		
Seeds, nuts		0.26			
Shell fish	0.28			0.23	0.24
Soda		−0.23		0.24	
Soup	0.44	0.32			
Sugar-sweetened beverages				0.37	
Sweet breakfast foods			0.39		
Tea			0.31		
Vegetable – cruciferous		0.59			
Vegetable – dark yellow		0.41			
Vegetable – green leafy	0.16	0.49		−0.22	0.48
Vegetable – other		0.48			
Vegetable – tomato		0.32			0.27
Vegetable mixed dishes	0.35	0.31			−0.25
Water		0.32			
Wine					0.36
Yogurt		0.31		−0.25	

Review of factor means across several demographic and socio-economic variables revealed group differences (Table 4). Older participants had lower scores on the Southern, Convenience, and Alcohol/Salads patterns and higher scores on the Plant-based pattern. Compared to white participants, black participants had lower scores on the Convenience, Sweets/Fats, and Alcohol/Salads patterns but the highest mean score on the Southern pattern (0.57 versus −0.29). Men had lower scores on the Plant-based pattern compared to women, but higher scores on the other four patterns. Those in the Southeastern US stroke belt had lower scores on the Convenience pattern (−0.07) and higher scores on the Southern pattern (0.10), which was an additional consideration when we named this pattern “Southern.” Participants with fewer years of education or lesser income had lower scores on the Convenience and Alcohol/Salads patterns and higher scores on the Southern pattern. The highest mean factor score observed across income category was in the Alcohol/Salads pattern, where those making more than $75,000 had a mean factor score of 0.42. In contrast, those making <$20,000 per year had a mean factor score of −0.39 on this pattern. The Southern pattern revealed converse associations with income: those making less than $20,000 had a mean score of 0.39 for the Southern pattern while those making more than $75,000 had a mean score of −0.32.

**Table 4:** **Mean factor scores for each dietary pattern derived in the REGARDS study**.

			Convenience		Plant-based		Sweets/fats		Southern		Alcohol/salads	
Variable	Level	*N*	Mean score	*p*-Value	Mean score	*p*-Value	Mean score	*p*-Value	Mean score	*p*-Value	Mean score	*p*-Value
Age	45–54	2567	0.33	<0.0001	−0.21	<0.0001	0.10	<0.0001	0.07	<0.0001	0.12	<0.0001
	55–64	8394	0.10		−0.05		−0.02		0.04		0.08	
	65–74	7090	−0.10		0.07		−0.02		−0.02		−0.03	
	75+	3585	−0.27		0.14		0.02		−0.10		−0.21	

Race	Black	7275	−0.21	<0.0001	0.12	<0.0001	−0.15	<0.0001	0.57	<0.0001	−0.34	<0.0001
	White	14361	0.10		−0.06		0.08		−0.29		0.17	

Region	Belt	12168	−0.07	<0.0001	−0.01	0.118	0.05	<0.0001	0.10	<0.0001	−0.05	<0.0001
	Non-belt	9468	0.09		0.01		−0.06		−0.13		0.07	

Gender	Female	12090	−0.13	<0.0001	0.10	<0.0001	−0.07	<0.0001	−0.11	<0.0001	−0.11	<0.0001
	Male	9546	0.16		−0.13		0.09		0.14		0.13	

Education	Less than high school	2090	−0.20	<0.0001	−0.08	<0.0001	0.09	<0.0001	0.54	<0.0001	−0.41	<0.0001
	High school graduate	5535	−0.07		−0.12		0.08		0.16		−0.13	
	Some college	5932	0.00		−0.03		0.01		0.02		−0.01	
	College graduate and above	8069	0.10		0.13		−0.09		−0.27		0.21	

Income	<$20k	3427	−0.13	<0.0001	0.00	0.016	0.04	<0.0001	0.39	<0.0001	−0.39	<0.0001
	$20k–$34k	5224	−0.10		−0.02		0.05		0.09		−0.12	
	$35k–$74k	6749	0.05		0.00		0.02		−0.08		0.09	
	$75k and above	3680	0.22		−0.01		−0.13		−0.32		0.42	
	Refused	2556	−0.07		0.06		−0.04		−0.05		−0.07	

## Discussion

Using EFA (PCA) and confirmatory analysis (CFA) in conjunction with comprehensive examination across population subgroups, we identified five dietary patterns in the REGARDS study. The empirically derived factors were distinct and interpretable and reflected meaningful patterns of dietary intake. They were also consistent across gender, race, and regional subgroups in the US. We confirmed the validity of the patterns by examining model fit using CFA in a split sample from the source population. Additional validity has also been demonstrated in a recent study in the REGARDS population, where 63% of the racial disparity in stroke risk for African Americans was mediated by the Southern pattern (18).

We derived two patterns that appear to have health promoting properties based on their food composition and the general nutritional epidemiologic literature. Specifically, both the alcohol/salads and plant-based patterns had high factor loadings for foods such as vegetables, leafy greens, nuts, seeds, and fish, which are beneficial for health (19–22). The alcohol/salads pattern also includes beer, wine, and liquor, which have been shown to be cardioprotective in moderation (23) although this association has not always persisted across race (24, 25). In contrast to the healthier items, however, processed meats, condiments, and added fats were also prominent in the alcohol/salads pattern. The oldest participants in REGARDS were most likely to adhere to this style of eating. Older individuals commonly have greater adherence to healthier dietary patterns compared with younger people (26, 27), perhaps due to chronic disease management. This finding may also be a survivor effect and the plant-based pattern might be associated with longevity. In contrast, the alcohol/salads pattern was more likely to be consumed by younger people. Both the alcohol/salads and plant-based patterns were associated with higher income. Our findings showing associations with sociodemographic characteristics such as age, gender, race, region, education, and income are consistent with other reports (28, 29).

We also identified three patterns that contain foods that are known to be associated with unfavorable health and disease outcomes. The sweets/fats dietary pattern had high factor loadings on many food groups that included added sugars. Recent studies indicate that the American diet is extremely high in added sugars (30). A pattern high in added sugar like this, one has been associated with excess energy intake and weight gain in some studies (31) but not others (17), thus further research is needed to test this hypothesis. Both the Southern and Convenience patterns shared a likeness with what other studies describe as “Western” (5, 7, 32–34), since both showed reasonably high factor loadings for red meat, processed meats, potatoes, refined grains, and fried foods. However, an important distinction observed in our study was the large factor loading for the organ meats food group that included liver, gizzard, neckbones, and chitlins. The pattern also included moderate loadings for shellfish and fried food (mainly fish and chicken), foods commonly consumed in the Southeastern US.

Overall, the five dietary patterns that we observed are consistent with those reported in a comprehensive review paper of 58 papers using factor analysis in diverse populations and settings (6). Though names vary somewhat across studies, healthful patterns high in plant foods and lean proteins and less healthful patterns high in meat, refined grains, and processed foods are particularly reproducible (1). Importantly, healthy dietary patterns have been consistently associated with a wide range of health and disease outcomes, including decreased inflammation (35) and depression (36, 37) and lower risks of heart disease (38), diabetes (39), colorectal cancer (40), breast cancer (41), and obesity (28, 33, 42, 43). We chose the name “plant-based” for our healthy pattern in this study since it is a more accurate reflection of its components and diets of differing food composition may still be considered healthy (44). It is not surprising that we derived a Southern pattern given the REGARDS study is focused in the Southern US. Yet, this pattern resonates well with the “Traditional” patterns often reported (17) since it essentially reflects a pattern commonly consumed by people living in the American South. Together, these results suggest that our patterns are consistent with the current literature, supporting the generalizability of major dietary patterns across diverse populations with understandable differences that reflect cultural differences in eating behavior.

Our study has several limitations. First, the FFQ was self-administered and we had differential return. The most highly educated participants were most likely to return the FFQ regardless of race, age, and region of the country. This could mean that we have an under-representation of people who have not graduated high school, and it could be due to different reading levels in those people with lower education. Future studies in REGARDS are planned to ascertain diet in participants who did not return FFQs to understand whether this difference in participation has a meaningful impact on studies of diet and disease. Second, REGARDS oversampled residents of Southern states and only enrolled black and white individuals. However, our patterns are similar to those observed in many other studies (45, 46) and we undertook rigorous methods that showed similarities across race, region, and gender subgroups, which showed that our patterns are stable. That said, our study excluded Hispanic Americans by design and several unique patterns have been observed in these populations (7). The Southern dietary pattern may have emerged partly due to the oversampling, and other patterns may be identified for other population subgroups. However, the greatest racial disparities in stroke are between black and white individuals, and our derived patterns have already been shown to be related meaningfully to outcomes such as stroke risk (18) as well as kidney disease (47).

Despite these limitations, our study has many strengths. First, REGARDS is a national bi-racial, geographically and economically diverse sample that is demographically similar to the US Census and the BRFSS, suggesting that our patterns may be representative of dietary patterns among Americans. Second, its large sample size allowed us to employ a split sample approach and subgroup analyses to examine the stability of our patterns. Third, we employed CFA in our study, a powerful method that further established the validity of the patterns derived using EFA. Together, our results further establish the validity and generalizability of major dietary patterns across diverse studies and populations and move forward our understanding of eating behavior, especially among those who reside in the Southern US who suffer disproportionately from chronic diseases.

In conclusion, we derived five meaningful dietary patterns in a large cohort of black and white Americans. We employed rigorous dietary pattern methods using both exploratory and CFA and found that our patterns were generalizable and stable across race, region, and gender subgroups. Our dietary patterns are consistent with the current evidence base and also showed interesting nuances given the REGARDS study focused on Southern Americans. Future research will examine associations with chronic diseases such as obesity, heart disease, and mortality to better understand the etiology of racial disparities in the US and help inform appropriate dietary guidance to diverse population subgroups.

## Conflict of Interest Statement

The authors declare that the research was conducted in the absence of any commercial or financial relationships that could be construed as a potential conflict of interest.

## Author Contributions

Dr. Suzanne E. Judd conducted research (hands-on conduct of the experiments and data collection), analyzed results, wrote paper, and had primary responsibility for final content. Mr. Abraham J. Letter performed statistical analyses, analyzed results, and wrote paper. Dr. James M. Shikany analyzed results and contributed to writing and editing the paper. Dr. David L. Roth performed statistical analyses and contributed to writing and editing the paper. Dr. P. K. Newby designed research (project conception, development of overall research plan, and study oversight), analyzed results, and wrote paper.

## Supplementary Material

The Supplementary Material for this article can be found online at http://www.frontiersin.org/Journal/10.3389/fnut.2014.00029/abstract
