# Supplementary figures and images for: Dietary Patterns Derived Using Exploratory and Confirmatory Factor Analysis are Stable and Generalizable Across Race, Region, and Gender Subgroups in the REGARDS Study
Source: Front Nutr. 2015 Jan 19;1:29. doi: 10.3389/fnut.2014.00029 (PMC4429641; doi:10.3389/fnut.2014.00029)

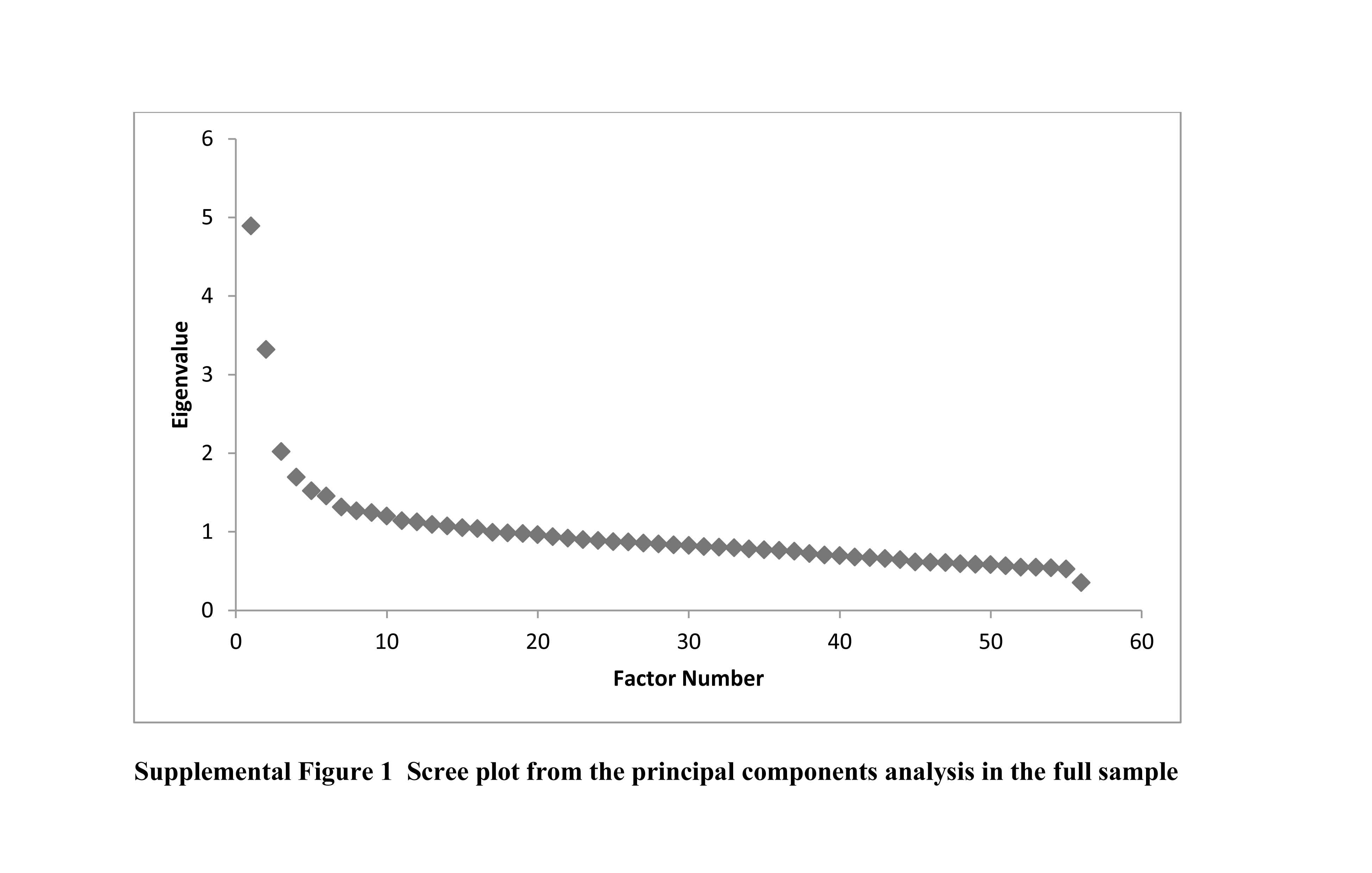

Supplement: Supplementary file 1 [file Image_1.TIF]
